# Postoperative Disparities Facing Patients Experiencing Homelessness: Opportunities for Advocacy From an Anesthesiology Perspective

**DOI:** 10.7759/cureus.89782

**Published:** 2025-08-11

**Authors:** Ashwin Mahesh, Sarah Chowdhury, Dana Gurvitch

**Affiliations:** 1 Anesthesiology, Weill Cornell, New York City, USA

**Keywords:** anesthesiology, eras, health equity, homelessness, postoperative disparities, public health

## Abstract

Patients experiencing homelessness (PEH) continue to face significant disparities across the world. Many of these disparities not only have downstream medical consequences but also housing-based consequences, including propagation of chronic homelessness. The postoperative period is a time of great medical vulnerability for PEH. Without targeted intervention, PEH are at risk for progression of disparate outcomes and chronic vulnerability. As integral players in the patient’s care team during this period, anesthesiologists are uniquely positioned to address these disparities and drive change for a critically marginalized population. In this review, we examine the current literature on disparities impacting PEH in the postoperative period, highlighting key areas such as complications, hospitalization length, readmission rates, acute emergency department (ED) utilization, cost to the health system, postoperative follow-up, and discharge processes. Despite the potential impact of these disparities, there is a paucity of literature specific to PEH. Additionally, given the variability of findings between institutions and across surgical specialties, further research characterizing disparities on a local level is required. Despite these gaps in knowledge, there is an urgent need for the creation and assessment of actionable interventions aimed at improving care for PEH. Here, we analyze themes from our review and propose interventions that anesthesiologists can lead, in collaboration with health system colleagues, to foster change.

## Introduction and background

Patients experiencing homelessness (PEH) continue to endure innumerable medical and social disparities globally. For many PEH, healthcare insecurity acts as a key factor in both the initiation and propagation of chronic homelessness. This relationship between healthcare and housing is often bidirectional, iterative, and cyclic in nature. For instance, a lack of stable access to healthcare frequently fosters initial housing instability and homelessness. Instability in housing further increases medical vulnerabilities, which in turn can augment housing insecurity (HI) [1]. Without targeted interventions directly addressing these medical vulnerabilities during critical periods, many PEH are at risk for progression of this cycle, accompanied by worsened housing outcomes, morbidity, and mortality.

Medical vulnerabilities for PEH occur across disciplines and are uniquely important when considering perioperative outcomes and risk stratification. PEH experience a disproportionate burden of chronic illness with increased rates of hypertension [2], diabetes-associated morbidity [3], obstructive lung diseases [4], cardiovascular disease and mortality [2], mental health and substance use conditions [5], HIV, hepatitis C [6], and more. This increased burden of undermanaged chronic medical conditions, paired with significantly decreased utilization of routine primary care [7,8], increased utilization of emergency departments (EDs) in the acute setting [9], and an up to 10 times greater standardized mortality rate compared to housed patients [10], creates a critical vulnerability for PEH in the perioperative period.

While there are notable disparities for PEH present in accessing surgical care during the preoperative and intraoperative periods, disparities in the postoperative period play an especially important role in compounding medical vulnerability. Within the postoperative period, significant disparities exist for PEH in surgical complications, morbidity, length of hospitalization, readmission rates, cost to the health system, acute ED utilization, access to post-surgical management, postoperative discharge procedures, and more [11]. Despite research highlighting these disparities within certain surgical fields, there has been limited focus on the compilation of data across specialties, with a notable dearth of literature related to the development of validated evidence-based interventions addressing disparities faced by PEH in the postoperative period. Therefore, when considering medical vulnerability in the context of unstable housing, the postoperative period presents a unique opportunity to not only improve medical outcomes but also directly intervene in cycles of homelessness.

**Figure 1:**
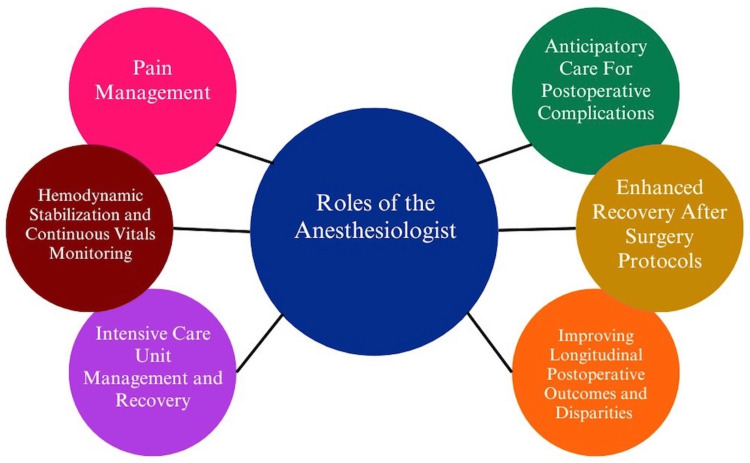
Postoperative roles of the anesthesiologist

Historically, surgeons, hospitalists, primary care physicians, outpatient specialists, and case managers have been identified as core facilitators in influencing change for patients leading up to and after operative discharge. However, with growing changes in the field of anesthesiology over the past few decades and rapid expansion of care outside of intraoperative management, anesthesiologists have taken on an increasingly important role in postoperative management. Roles such as postoperative acute and chronic pain services, intensive care unit (ICU) management, remote continuous vital monitoring, anticipatory care for postoperative complications, and the creation of interventions aiming to reduce failure to rescue (FTR) rates and protocols to enhance recovery after surgery (ERAS) demonstrate a few ways that the role of the anesthesiologist has expanded to encompass outcomes in the postoperative period and beyond (Figure 1) [12]. As this role continues to expand, anesthesiologists are faced with a growing frontier of opportunities to innovate and drive change in areas with unanswered postoperative gaps. One such area is the disparities that PEH face in the postoperative period. As we describe here, the landscape of unanswered postoperative disparities for PEH is expansive. Recognizing that the task of answering these disparities is immense and requires collaboration from multiple specialties and disciplines, we propose a role that anesthesiologists can play in leading this collaboration with primary care teams. As patient advocates throughout the perioperative experience and with their key connections with stakeholders across disciplines, anesthesiologists hold a unique positioning to organize and advocate for change for PEH. Here, we aim to provide an overview of literature describing the evolving landscape of postoperative disparities for PEH, discuss potential next steps for members of the care team, and describe a role for anesthesiologists to help drive change.

## Review

Methods

A literature search for this narrative review was conducted between January 1, 2010, and April 30, 2025, within numerous databases, including PubMed, Google Scholar, Web of Science, and SCOPUS. The search approach was modified to include keywords and phrases capturing the breadth of literature related to postoperative disparities in patients experiencing homelessness (PEH). Medical Subject Headings (MeSH) phrases and Boolean operators were utilized to optimize the search strategy. An example of a primary search query utilized was “postoperative” AND “homelessness”, with applicable synonyms substituted as needed. As themes were deciphered with initial broad searches, more directed queries such as “surgical complications” AND “homelessness” were utilized. Studies were included if they were peer-reviewed, topically relevant and impactful, readily accessible, written in English, and within the time frame evaluated. Studies not addressing postoperative disparities in PEH, not driven by original data, with limited full-text access, or outside of the time frame evaluated were excluded. Each study was carefully reviewed to assess the potential risk of bias before inclusion, including study design, variable definitions of homelessness, and variations in outcome measures. To limit selection bias, studies were independently reviewed by two authors to evaluate adherence to selection criteria. Twenty-two studies that received independent author consensus were included, while studies not receiving independent consensus were excluded.

Disparities in post-surgical complications

A common framework utilized by anesthesiologists and surgeons to investigate postoperative complications is the modified Clavien-Dindo system, an evidence-based framework that grades complications from I to V based on severity [13]. PEH experience clear disparities in rates of post-surgical complications and outcomes ranging in severity from grade II to grade V. There is a dearth of research investigating rates of grade I complications, such as minor pain, infection not requiring medication, and minor suture complications in PEH. Grade II complications are more severe than grade I and require pharmacotherapy for management outside of antiemetics, antipyretics, analgesics, electrolyte repletion, and diuretics. Over the past few decades, evidence has surfaced related to grade II complications in populations experiencing homelessness. For instance, one recently published study investigating a population of patients with hip fractures requiring surgical repair found that PEH had significantly higher rates of 90-day postoperative urinary tract infections (UTIs) (odds ratio (OR): 1.37, 95% confidence interval (CI): 1.19-1.58), pneumonia (OR: 1.49, 95% CI: 1.28-1.74), and surgical site infections (SSIs) (OR: 2.03, 95% CI: 1.62-2.54). This same study conducted a multivariate logistic regression and identified experiencing homelessness as an independent risk factor for developing UTIs and SSIs within 90 days of repair surgery. This study also identified increased odds of respiratory failure (OR: 1.33, 95% CI: 1.16-1.52) and acute renal failure (OR: 1.36, 95% CI: 1.18-1.56) in the 90-day postoperative period for PEH [14]. It is important to note that cases of respiratory failure requiring intubation and acute renal failure requiring dialysis are considered higher-risk complications than grade II (grade IV). Given that these qualifiers were not reported in this study, we have included these findings under discussion of grade II and grade IV complications.

Another study investigating postoperative morbidity in PEH in North America found a trend toward increased SSIs in unhoused patients (0.3% in unhoused patients and 0.1% in housed patients, p=0.02) [15]. This finding of increased SSIs was also identified in a study investigating the impact of housing insecurity (HI) on postoperative outcomes in veterans undergoing abdominal aortic aneurysm repair (10.3% in veterans with HI and 1.8% in veterans without, p=0.012) [16]. However, it is important to note that these disparities in grade II complications have not been universally replicated across all surgical operations and may vary between institutions and specialties. For instance, one study investigating care for PEH who received oncologic surgery found no significant differences in postoperative complications from housed populations, including grade II complications, despite PEH being more likely to undergo unplanned surgery (OR: 5.17, 95% CI: 3.00-8.92) and having increased odds of readmission (OR: 1.81, 95% CI: 1.07-3.05) [17]. Similarly, another study investigating care for PEH undergoing emergency surgery admission found no significant differences in complications, including grade II complications, from housed populations, despite having lower odds of outpatient follow-up (OR: 0.54, 95% CI: 0.35-0.85) and higher emergency department (ED) utilization (OR: 2.72, 95% CI: 1.78-4.14) [18]. Variability in these findings suggests that disparities in postoperative complications may be nuanced, specialty-specific, and even operation-specific. Given a paucity of data specific to many surgical subspecialties, these findings warrant expansion of research initiatives to better characterize next steps for needed interventions.

Grade III complications are those that require procedural intervention, including surgical, radiology-based, and endoscopic procedures. Over the last few decades, numerous studies have characterized significant disparities in grade III complications for PEH. One study investigating care for foot osteomyelitis found that experiencing homelessness was a moderate risk factor for surgical treatment failure (hazard ratio: 9.5, 95% CI: 3.3-27.3) and subsequent leg amputation (hazard ratio: 11.1, 95% CI: 2.5-50.5) [19]. An additional study exploring outcomes in patients with ankle fractures who underwent open reduction and internal fixation found that PEH had significantly higher odds of needing repeated open reduction and internal fixation (adjusted OR (aOR): 1.16, 95% CI: 1.00-1.35, p=0.045), amputation (aOR: 1.59, 95% CI: 1.08-2.27), and irrigation and debridement (aOR: 1.22, 95% CI: 1.08-1.37) [20]. A separate study investigating the influence of housing status on operations over a decade at a tertiary center found that PEH had significantly increased rates of in-hospital reoperations, although adjusting for demographic factors, comorbidities, and surgical indication diminished this finding [21]. Although not exclusively investigating PEH, a second study evaluating the impact of socioeconomic deprivation on post-surgical outcomes in ankle fractures found similar findings with greater socioeconomic disparity increasing risk for further irrigation and debridement (OR: 2.14, 95% CI: 1.25-3.67) and amputation (OR: 3.56, 95% CI: 1.01-12.4) [22]. An additional study found that patients readmitted post-percutaneous coronary intervention (PCI) were more likely to have a history of homelessness, compared to those who were not readmitted (3.2% versus 1.6%, p=0.007) [23]. Given the increased proportion of readmissions after PCIs that involve radiological intervention, we have included this finding here as potential evidence for disparity in grade III complications, acknowledging the need for expanded research in this area [24]. From these studies, it is clear that there are significant intervenable disparities present in grade III postoperative complications for PEH that impact not only repeat procedural intervention rates but also morbidity.

Grade IV complications are those that are life-threatening and require ICU or intermediate management due to severity. Grade V complications are the most severe complications that ultimately lead to patient mortality. Recent literature has described disparities in grade IV and V complications for PEH. One study that was previously described investigating patients with hip fracture necessitating surgical repair found that in the 90-day postoperative period, PEH had increased odds of developing sepsis (OR: 1.70, 95% CI: 1.44-1.99), respiratory failure (OR: 1.33, 95% CI: 1.16-1.52), and acute renal failure (OR: 1.36, 95% CI: 1.18-1.56) [14]. Although these conditions do not always necessitate ICU management, they are often indications for escalation of treatment. For instance, septic shock, respiratory failure requiring intubation, and acute renal failure requiring dialysis are all considered grade IV complications. Given that the analysis from this paper does not include further categorization, we are including these findings under grade IV complications in addition to grade II.

When discussing grade V complications, there has been preliminary evidence supporting increased postoperative vulnerability for PEH with respect to mortality in some fields. For instance, one study investigating mortality in veterans following coronary artery bypass grafting surgeries found that homelessness was an independent predictor of 30-day postoperative mortality (OR: 6.49, 95% CI: 3.39-12.45) [25]. Another study previously described investigating postoperative outcomes at a tertiary center reported that PEH had a higher one-year mortality after surgery than housed counterparts, although this finding was reduced with adjustment for demographic factors, comorbidities, and surgical indications [21]. Another study described previously found that veterans experiencing housing instability had lower median postoperative survival after abdominal aortic aneurysm repair compared to those who were housing stable, although no significant difference was noted in 30-day mortality [16]. It is important to note that, despite these findings, studies in other surgical fields have demonstrated no increase in postoperative mortality for specific surgeries and institutions. For instance, one previously described study investigating oncologic surgery outcomes found that PEH had comparable postoperative mortality and morbidity compared to housed counterparts [17]. Another study evaluating outcomes in lower extremity bypass procedures found no significant difference in one-year mortality or amputation rates, despite increased burden of procedures performed in PEH [26]. Variances in data for postoperative mortality for PEH across institutions and surgeries further accentuate the need for the expansion of specialty-specific research to help guide outcomes. Table 1 presents examples of disparities in PEH classified according to the modified Clavien-Dindo system.

**Table 1:** Examples of disparities in postoperative complications observed in patients experiencing homelessness, classified according to the modified Clavien-Dindo system PEH: patients experiencing homelessness, UTI: urinary tract infection, ORIF: open reduction and internal fixation, CABG: coronary artery bypass graft Table adapted using the modified Clavien-Dindo classification system [13]

Grade	Definition	Disparities in postoperative complications in PEH
I	Any postoperative complication not requiring pharmacological, surgical, endoscopic, or radiological treatment	-
II	Complications requiring therapeutic regimen outside antiemetics, antipyretics, analgesics, diuretics, electrolytes, and physiotherapy; includes blood transfusion, total parenteral nutrition	Increased odds of 90-day postoperative UTI, pneumonia, and surgical site infections [14]; increased rates of surgical site infections [15,16]
III	Complications requiring any procedural intervention (i.e., surgical, radiological, and endoscopic)	Increased risk of treatment failure and amputation in osteomyelitis [19]; increased odds of amputation, irrigation and debridement, and repeat ORIF in ankle fracture [20]
IV	Critical complication requiring intermediate or intensive care management (including central nervous system complications)	Increased odds of 90-day postoperative respiratory failure, sepsis, and acute renal failure [14]
V	Complications leading to death	Increased odds of 30-day postoperative mortality following CABG [25]

Disparities in postoperative hospitalization length and readmission

In addition to disparities in complications, PEH face increased hospitalization length and readmission rates following surgeries (Table 2). For instance, a previously described study found that PEH had a nearly 68% increase in adjusted length of stay (95% CI: 42.0-98.2%) and higher odds of readmission (OR: 1.81, 95% CI: 1.07-3.05) following oncologic surgery [17]. Similarly, another study found that within surgical admissions, length of stay was increased for PEH, significantly increasing the costs of admissions [27]. A separate study evaluating outcomes in patients with burns severe enough to necessitate inpatient management found that PEH had significantly longer length of stays, despite receiving fewer operations and being more likely to leave against medical advice [28]. Similarly, a previously described study found that homelessness is more prevalent in patients experiencing readmission following PCI [23]. Further, among veterans receiving orthopedic, vascular, and general surgery, PEH had higher odds of readmission than housed counterparts (OR: 1.43, 95% CI: 1.30-1.56) with risk factors including elevated American Society of Anesthesiologists classification score (OR: 1.86, 95% CI: 1.30-2.68), alcohol abuse (OR: 1.45, 95% CI: 1.15-1.84), and discharge to the community (OR for those discharged other than to the community: 0.57, 95% CI: 0.44-0.74) [29]. A study investigating emergency surgery admissions also found that PEH had greater odds of readmission (OR: 1.87, 95% CI: 1.09-3.19) [18]. Taking this literature into account, there is a strong body of evidence demonstrating increased length of stay and readmission rates for PEH across many surgical specialties. However, there are still significant gaps in knowledge related to understanding critical risk factors that contribute to these disparities. Moreover, the creation of validated risk calculators may be beneficial in identifying PEH at greater risk of readmission and delineating subpopulations that would benefit from targeted intervention early in the hospital course. In the following discussion, we describe health system costs, one metric that may be reduced with direct intervention aimed at improving length of stay and readmission rates.

**Table 2:** Disparities in postoperative hospitalization length and readmission PEH: patients experiencing homelessness, PCI: percutaneous coronary intervention

Study	Findings
Silver et al. (2024) [17]	Increased adjusted length of stay and higher odds of readmission for PEH following oncologic surgery
Hwang et al. (2011) [27]	Within surgical admissions, increased length of stay for PEH
Kiwanuka et al. (2019) [28]	Greater length of stay in PEH admitted inpatient for burns, despite receiving fewer operations
Wasfy et al. (2015) [23]	Homelessness was more prevalent in patients readmitted within 30 days of PCI
Titan et al. (2018) [29]	Higher odds of readmission in veterans experiencing homelessness who received orthopedic, vascular, and general surgery
Smith et al. (2024) [18]	Higher odds of readmission for PEH in emergency surgery admissions

Emergency department utilization and cost to the health system

PEH are less likely to have longitudinal follow-up with primary care [7,8] and more likely to utilize ED services [9]. As established here, the postoperative period is an especially critical time for PEH with increased burden of complications, readmissions, and length of stays. This added vulnerability, paired with a lack of stable follow-up with primary care, creates a period of increased utilization of ED care following operative discharge for many PEH (Table 3). For instance, one study investigating ED utilization after open reduction and internal fixation or intramedullary nailing for lower extremity fractures found that PEH had greater odds of increased postoperative ED utilization (OR: 3.91, 95% CI: 1.53-9.98) [30]. Another study evaluating outcomes for PEH with orthopedic traumas found that PEH had significantly more ED visits compared to housed counterparts following discharge [31]. A separate study previously described also found that PEH undergoing emergency surgery admissions had higher odds of ED utilization upon discharge (OR: 2.72, 95% CI: 1.78-4.14) [18]. Notably, PEH have significantly increased ED utilization following hospital encounters for physically traumatic injuries, including those requiring medical or surgical management [32]. The growing body of evidence describing increased utilization of ED services following operative discharge indicates potential vulnerability in post-surgical continuity of care for PEH. We discuss this further in the discussion section below.

**Table 3:** Disparities in postoperative emergency department utilization in PEH PEH: patients experiencing homelessness, ED: emergency department

Study	Findings
Rafael Arceo et al. (2018) [30]	Greater odds of increased postoperative ED utilization after open reduction and internal fixation or intramedullary nailing for lower extremity fractures
Kay et al. (2014) [31]	Significantly greater number of ED visits following discharge for orthopedic trauma
Smith et al. (2024) [18]	Higher odds of ED utilization after discharge for emergency surgery admissions
Beaulieu-Jones et al. (2025) [32]	Significantly increased ED utilization following hospital encounters for physically traumatic injuries, including those requiring medical or surgical management

Increased ED utilization also raises overall cost to both PEH and the health system. One factor accounting for this cost is variability in the cost of equivalent services when delivered in the ED versus in internal medicine or primary care settings. For instance, a study found that the markup ratio, a measure of excess charge, for ED services was substantially higher than for internal medicine across multiple hospitals. This indicates a significantly higher cost burden to patients, especially patients without insurance coverage [33]. This trend of disparate ED costs has drastically increased over time [34]. With growing evidence for elevated per-visit costs within EDs, it is apparent that trends of increased postoperative ED utilization in PEH have the ability to exacerbate financial strain in an already financially vulnerable population. These financial costs also apply on a health system level. One study analyzing ED spending from 2006 to 2016 found that ED spending increased from $79.2 billion to roughly $136.6 billion, with costs per ED visit steadily increasing from $660 to $943 over this period [35]. With these trends contributing to gradually increasing health system spending, it is more important than ever to address potential drivers such as lack of access to stable outpatient care following surgery for PEH.

In addition to increased postoperative ED utilization, there are numerous studies relating experiencing homelessness to increased cost of surgical admission (Table 4). One previously described study found that PEH had an increased cost of surgical admission, thought to be driven in part by the disparity of length of stay established in prior sections [27]. Another previously cited study on oncologic surgery outcomes found that the adjusted cost of admission in PEH was 32.7% (95% CI: 14.5%-53.9%) higher than housed counterparts [17]. This finding is further supported by research in burn admissions that found that PEH had significantly higher costs of admission [28]. A separate study evaluating the impact of housing instability on surgical conditions where timely access is critical found that patients who were housing unstable had significantly higher costs per admission and length of stay, despite having lower odds of complications [36]. Notably, within the United States, this finding of increased cost of surgical admissions is further exacerbated by the lack of Medicaid expansion, with states without Medicaid expansion having significantly increased costs for emergency general surgery for PEH [37].

**Table 4:** Increased costs of admission PEH: patients experiencing homelessness

Study	Findings
Hwang et al. (2011) [27]	Increased cost of surgical admissions for PEH
Silver et al. (2024) [17]	Increased adjusted cost of admission for PEH receiving oncologic surgery
Kiwanuka et al. (2019) [28]	Increased cost of admission for PEH admitted for burns
Evans et al. (2025) [36]	Increased cost of admission and length of stay in patients with housing instability

A potential driver of increased postoperative readmissions, ED utilization, cost to the health system, and complications in PEH is a lack of stable postoperative follow-up. One study described here found that PEH had lower odds of outpatient follow-up after emergency surgery admissions (OR: 0.54, 95% CI: 0.35-0.85) [18]. Another study investigating orthopedic traumas found that PEH had fewer outpatient orthopedic clinic visits than housed patients, despite PEH treated with operative management having greater rates of follow-up than PEH without [31]. Little has been described as to whether these disparities in postoperative follow-up are related to access, utilization, or multifactorial. Nevertheless, there has been data showing promising postoperative outcomes and follow-up in health systems with robust medical and social services [38], indicating a prominent role for system-level interventions to address this disparity. Notably, within the United States, PEH who required emergency surgery had lower odds of receiving supportive community healthcare post-discharge in non-Medicaid expansion states, also suggesting potential areas for structural-level interventions [37].

Disparities in postoperative follow-up and discharge

When discussing post-discharge care for PEH, collaboration with social work and community support is imperative, especially during periods of vulnerability such as the postoperative context. One important metric when considering readmission risk is discharge location. A study investigating postoperative readmissions in veterans found that discharge to locations other than directly back to the community was associated with significantly decreased odds of readmission (OR: 0.57, 95% CI: 0.44-0.74) [29]. Another previously described study evaluating operative outcomes in veterans found promising rates of postoperative follow-up and housing-based outcomes in a case series of 33 patients, of which 30 patients were discharged to a facility or shelter [38]. In a study investigating outcomes following surgery for malignancy, PEH had higher odds of facility discharge compared to housed patients (aOR: 5.89, 95% CI: 3.50-9.78) [17], with PEH who were discharged directly to the community having increased odds of readmission compared to housed patients (OR: 1.81, 95% CI: 1.07-3.05). Further research is required to understand drivers of this disparity and the relationship between discharge, postoperative follow-up, and readmissions. In the sections below, we describe an applicable framework for structuring health system and community-level interventions targeting postoperative follow-up outcomes.

Finally, it is important to acknowledge the role that trust may play in compounding vulnerability in the postoperative period. Many PEH have faced stigma and trauma within health systems. As such, PEH can have significantly reduced trust in medical institutions and care providers, making the physician-patient relationship a crucial gateway for longitudinal care [39]. With this in mind, PEH also have increased rates of discharge against medical advice during the postoperative period [28]. One factor that may contribute to this is reduced trust in provider teams, although little has been described investigating root causes. Further research is necessary to characterize this disparity and understand its relation to other described disparities in the postoperative period.

Discussion and opportunities for advocacy in anesthesiology

In the sections above, we have provided an overview of disparities that PEH face in the postoperative period. Here, we describe key themes and takeaways to help guide next steps to support PEH. With respect to postoperative complications, there is a clear need for replication of studies on a local level. As accentuated in our discussions of grade II and grade V complications, there is variability in the identification of complications across surgical specialties and institutions. These findings suggest that disparities in complications may be varied by surgical subspecialty, surgery type, and environmental factors present at the institution level. Replication of this work across different specialties and on a local level will allow for further characterization of driving factors underlying disparities and help guide intervention development.

Moreover, we have described evidence supporting increased length of stays in PEH within postoperative contexts. Despite this, there is a gap in the literature evaluating drivers of increased length of stay specific to the postoperative period. Outside of postoperative literature, there is evidence suggesting that length of stay in hospitalized PEH is significantly influenced by comorbidity with mental health disorders, infectious diseases, and duration of homelessness [40]. These three factors may be reliable targets for future intervention development, although investigation specifically within postoperative admissions is necessary.

We have further described evidence for increased postoperative readmission risk in PEH. As detailed previously, there is initial evidence suggesting an elevated American Society of Anesthesiologists classification score, alcohol abuse, and discharge to the community as three risk factors for postoperative readmission in PEH [29]. However, similar to length of stay, there is a dearth of information on additional risk factors governing this disparity. Outside of postoperative literature, factors such as comorbidity with substance use disorders, obesity, epilepsy, arthritis, HIV/AIDS, depression, and chronic kidney disease have been linked to shorter times to readmission in PEH [41]. Further research is required to evaluate risk factors specific to postoperative admissions and to develop risk stratification tools that can be employed early in the operative course.

Furthermore, evidence supporting increased postoperative ED utilization in PEH may accentuate a known system-level vulnerability of reduced utilization of primary care in PEH [7,8]. Studies have demonstrated that in times of increased medical vulnerability for PEH, such as during extreme temperature fluctuations, ED utilization is often increased [42,43]. A similar finding may explain the trends observed in postoperative ED utilization in PEH, with increased medical vulnerability driving ED utilization during this critical period. Future research that stratifies the acuity of these postoperative ED visits will be helpful in assessing the potential role of community-based primary care interventions in improving timely access to care.

Finally, we describe significant disparities in postoperative follow-up and discharge outcomes for PEH. Although it is unknown whether reduced postoperative follow-up is driven by difficulty in accessing postoperative clinics from the community, communication barriers, trust factors, or more, there have been health systems that have demonstrated promising follow-up through robust medical and social services post-discharge [38]. At our institution in New York City, the creation of a medical clinic directly within a food pantry accessed by many PEH has facilitated timely access to needed medical management [44]. Similarly, postoperative clinics built directly within the community may improve postoperative follow-up and mitigate trends related to readmissions, increased ED utilization, and cost to the health system.

As demonstrated here, the scope of postoperative disparities for PEH is expansive and requires collaboration across multiple specialties to answer. Anesthesiologists, with their evolving role within postoperative contexts and key connections with stakeholders, are uniquely positioned to influence change.

Anesthesiologists have drastically changed the landscape of disparities research and interventions in the perioperative period. One way this has been approached is through the creation of enhanced recovery after surgery (ERAS) protocols, standardized protocols aimed at augmenting outcomes, improving quality of care, and decreasing costs in the perioperative period [45]. These protocols have demonstrated the ability to reduce postoperative disparities, including those related to length of stay and complications [46,47]. Moreover, these protocols have been adapted to investigate and answer disparities that marginalized populations face [48]. One potential pathway for anesthesiologists to influence change for PEH, specifically with regard to length of stay and postoperative complications, is through the continued creation and adoption of standardized surgery-specific ERAS protocols. However, there has been an identified gap in the study of variables related to social determinants of health, including housing, within ERAS protocols [49]. This has led to gaps in knowledge on the effectiveness of specific protocols in reducing the disparities that we have described that PEH experience. By including housing status as a variable in the study of ERAS protocols, we can better understand these disparities and modify existing protocols to target each disparity individually.

Further, another way that anesthesiologists have increased accessibility to care in postoperative contexts is through innovations in telemedicine [50]. Specifically, anesthesiologists have developed remote vitals monitoring strategies in efforts to reduce complication rates following high-risk surgeries [51]. With the widespread growth of these strategies, there may be a role for the inclusion of remote monitoring in standardized postoperative protocols for PEH with high-risk comorbidities or known barriers to accessing follow-up care. Further research is required to study the feasibility and effectiveness of such strategies in answering the disparities discussed related to complications, follow-up, and ED utilization. Additionally, anesthesiologists have developed telemedicine programs to expand the reach of preoperative evaluations, often including supporting staff (nurses, physician assistants, and certified registered nurse anesthetists) to improve feasibility [52,53]. For PEH, early preoperative optimization using telemedicine presents an opportunity to both target known postoperative disparities prior to hospital course and facilitate early connection with hospital social workers to coordinate discharge and follow-up care. Establishing this infrastructure at accessible community-based sites is a strategy that can be employed to combat barriers to digital access.

Finally, outside of the scope of daily practice, anesthesiologists can advocate for system and structural level changes. Advocacy for improved facility discharge access and the development of alternate level discharge sites (e.g., respite care and health system-funded intermediate housing) may drive change in noted disparities related to discharge to the community and readmission risk. It is important to note potential obstacles to these efforts within the contexts of housing policy, institutional funding, and local resources. On a structural level, advocacy for housing-first interventions and expansion of supportive housing are ways to reduce core vulnerability that propagates homelessness [54].

## Conclusions

Here, we have outlined significant disparities that PEH face in the postoperative period with respect to complications, hospitalization length, readmissions, ED utilization, cost to the health system, follow-up, and discharge. We then analyzed this data, highlighted gaps present in current knowledge, and proposed important areas of future study. Finally, we described potential interventions that anesthesiologists can lead in collaboration with health system colleagues to drive change for this vulnerable population. Structured investigations evaluating social determinants of health, including housing, as variables within the study of ERAS protocols, may uncover targets for tailored intervention development for PEH. Advances in telemedicine and remote monitoring provide a promising avenue for both preoperative and postoperative optimization. Acknowledging challenges at the institutional, community, and policy levels, there is potential to drive lasting change for PEH in the postoperative period.
